# Protective properties of *Rydingia persica* in reproductive complications induced by diabetes in male rats: An experimental study

**DOI:** 10.18502/ijrm.v20i2.10504

**Published:** 2022-03-21

**Authors:** Fereshteh Ostovan, Ali Gol, Abdolreza Javadi

**Affiliations:** ^1^Department of Biology, Faculty of Science, Shahid Bahonar University of Kerman, Kerman, Iran.; ^2^Pathology Department, Shahid Beheshti University of Medical Sciences, Tehran, Iran.

**Keywords:** Diabetes mellitus, Rydingia persica, Oxidative stress, Reproductive, Testosterone.

## Abstract

**Background:**

In Iranian traditional medicine, *Rydingia persica *(R.P) is commonly used to treat diabetes mellitus (DM).

**Objective:**

We assessed the protective effects of R.P against testis and epididymis oxidative stress and the hormonal changes induced by DM.

**Materials and Methods:**

Forty male Wistar rats (12 wk old) weighing 230-270 gr were divided into five groups (n = 8/each): 1. Control (C); 2. diabetic (D); 3. diabetic + R.P200 (D+R200); 4. diabetic + R.P400 (D+R400); and 5. diabetic + R.P600 (D+R600). Groups C and D received 2 ml of normal saline orally daily for two wk and groups D+R200, D+R400, and D+R600 received 200, 400, and 600 mg/kg body weight of R.P powder, respectively, orally daily for two wk. DM was induced by a single intraperitoneal injection of streptozotocin at 60 mg/kg body weight. We assessed malondialdehyde, glutathione peroxidase, glutathione reductase, superoxide dismutase, catalase, hydrogen peroxide, and glutathione in both the testis and epididymis and also the histological changes of the testis.

**Results:**

Diabetic rats showed a significantly increased and decreased level of oxidant and antioxidant factors, respectively, and a significantly lower level of serum testosterone and luteinizing hormone than the control group. In the histological study of the testis, deteriorations were observed. Treatment with R.P reversed these changes toward the state of the control group with the highest effectiveness shown by group D+R600.

**Conclusion:**

The data obtained suggest that R.P powder has antioxidant effects on testis and epididymis tissues in diabetic rats and that it improves histological testicular structure in diabetics. It can also correct testosterone and luteinizing hormone changes induced by DM

## 1. Introduction

Diabetes mellitus (DM) is diagnosed based on a high serum glucose level due to a lack of insulin secretion (1), absence of insulin receptor responsiveness, or both. Overall, DM is one of the most important causes of death in developed countries and is responsible for 5% of deaths worldwide (2). DM can lead to many structural and functional effects such as neuropathy, nephropathy, retinopathy, and also disorders of the reproductive system (3, 4). Clinical and experimental investigations have revealed major changes in semen volume, spermatogenesis, sperm count, sperm motility, penile erection, ejaculation, serum luteinizing hormone (LH), follicle stimulating hormone, and testosterone levels as well as onset of puberty in diabetic subjects (5-7).

Oxidative stress (OS) is an important factor induced by hyperglycemia at the beginning and progression of DM complications. One of the main factors affecting the reproductive system in DM is the increased production of reactive oxygen species (ROS), which leads to protein and lipid oxidation (8). The balance between ROS and their scavengers is controlled tightly under physiological conditions. Defects in this balance can occur in metabolic disorders such as DM causing OS in the male reproductive system which can lead to a range of problems. Testicular oxidative damage can lead to biochemical and hormonal changes followed by a decrease in sexual libido and behavior (9-11).

In recent decades, some studies have shown the therapeutic effects of medicinal plants to control DM by modulating carbohydrate metabolism, repairing the pancreatic beta cell function and release of insulin, improving glucose utilization, and through their antioxidant properties (10, 12).

The aerial parts of *Rydingia persica *(Burm.f.) Scheen & V.A.Albert (R.P) - also known as *Otostegia persica* - have long been used as a medicinal herb in Iran and elsewhere in the Arab world and wider Asia as a way to alleviate various disorders (13). R.P is a medicinal plant containing polyphenolic compounds. These compounds are extensively distributed in different parts of the plant (14). R.P has some biological effects such as antioxidative, antidiabetic, anticancer, antiarthritic, and antimalarial effects (15, 16). Aerial parts of R.P contains components such as flavonoids, steroids, tannins, triterpenoids, and some essential mineral elements. Most of the mentioned compounds have been demonstrated to possess strong antioxidant activity (17). Polyphenols may exert their protective effects through direct and indirect antioxidant activity. The direct effects are the result of in vitro metal chelation, ROS scavenging, lipid peroxidation inhibition, and hydroperoxide formation reduction. The indirect effects are the result of modulation of pathways in cell signaling and gene expression, adjustment of defense enzymes, and alteration in nuclear histone acetylation (18).

This plant has been used for many varieties of disorders, including DM, but no studies have been done on its use for the reproductive complications of DM. We therefore decided to investigate the protective properties of the aerial parts of R.P against reproductive damage induced by DM by assessing the oxidative status and hormonal and histopathological changes in male rats.

## 2. Materials and Methods

This experimental study was carried out in the Department of Biology, Shahid Bahonar University of Kerman in November 2018.

### Chemicals and devices

Streptozotocin (STZ, 18883-66-4), 5', 5'-dithiobis-2-nitrobenzoic acid (DTNB, 69-78-3), β-nicotinamide adenine dinucleotide 2´-phosphate (NADPH, 2646-71-1), riboflavin (83-88-5), L- glutathione oxidized (GSSG, 27025-41-8), glutathione reductase (9001-48-3), and xylol (1082972500) were purchased from Sigma Aldrich, USA. Potassium iodide (KI, 1.05040.1000), dipotassium hydrogen phosphate (K ${}_{2}$ HPO ${}_{4}$ , 1.05101.1000), potassium dihydrogen phosphate (KH ${}_{2}$ PO ${}_{4}$ , 1.04873.1000), sodium dihydrogen phosphate (NaH ${}_{2}$ PO ${}_{4}$ , 1.06346.1000), hydrogen peroxide (H ${}_{2}$ O ${}_{2}$ , 1.08600.1000), meta-phosphoric acid (1.00546.0100), eosin (1.15935.0025), trichloroacetic acid (TCA, 1.00807.0250), and glutathione (GSH, 1.04090.0005) were purchased from MERCK, Germany. Hematoxylin was purchased from Pouya Abzar (Azma, Iran) and formalin (37%, A-330) from Atlas Shimi, (Iran). The spectrophotometer (Cary 50) was purchased from Varian (Australia).

### Preparation of *Rydingia persica* powder

The aerial parts of the R.P were collected from Birk Mountain, Saravan, in Sistan and Baluchestan province of Iran, and were taxonomically identified and approved by Dr. Mirtadzaddini, Biology Department of Faculty of Science, herbarium number (2388). The name of the plant was checked with dow.theplantlist.org. The R.P was powdered in an electrical grinder.

### Animals

In this experimental study, 40 adult male Wister rats (230-270 gr, 12-wk old) were obtained from the animal house of Shahid Bahonar University of Kerman and were maintained under standard colony conditions with a 12-hr light/dark cycle at constant room temperature (23 ± 2 C) with free access to food and water.

### Experimental design 

Streptozotocin (STZ) (Sigma-Aldrich, USA; 60 mg/kg body weight) was used for DM induction. STZ, dissolved in cold normal saline, was injected intraperitoneally. Seventy-two hr thereafter, the fasting blood glucose level was measured by a glucometer (Arkray Glucocard 01-mini glucometer, Japan), and the rats with a blood glucose over 300 mg/dl were allocated as diabetics. The study period was considered as 14 days according to a previous study (19).

The rats were divided into five groups (n = 8/each) and were fed orally for two wk:

Group C: Control rats, animals received normal saline.

Group D: Diabetic rats, animals received normal saline.

Group D+R200: Diabetic rats, received 200 mg/kg body weight R.P powder dissolved in normal saline.

Group D+R400: Diabetic rats, received 400 mg/kg body weight R.P powder dissolved in normal saline.

Group D+R600: Diabetic rats, received 600 mg/kg body weight R.P powder dissolved in normal saline.

On the 14 ${}^{\text{th}}$ day of the experiment, the animals were decapitated, and blood serum for hormonal changes was collected. The testes and epididymis were removed, weighed, and then frozen until OS assay.

### Serum preparation 

Blood samples were collected and centrifuged at 3500 rpm for 15 min to obtain serum samples and then were stored at -20 C until biochemical analysis. Commercially available ELISA kits were used to estimate hormones and LH (Testosterone ELISA kit-DKO002, Diametra, Italy and rat LH ELISA kit-ER1123, Fine test, China, respectively). Assays were performed according to the manufacturers' protocols.

### OS markers measurement

To measure the malondialdehyde (MDA) concentration, 0.2 gr of the desired tissue was homogenized in 5 ml of 0.1% trichloroacetic acid and the obtained solution was centrifuged for 10 min at 10,000 g and 4 C. Then, 1 ml of the supernatant was added to 20% trichloroacetic acid solution with 0.5% thiobarbituric acid. Finally, the mixture was heated for 30 min at 95 C and then cooled quickly in ice, and again was centrifuged for 10 min at 4000 g. Finally, the absorbance at 532 nm was read (20).

The amount of reduced glutathione (GSH) was measured using the total and oxidized glutathione (GSSG). Half a gr of the target tissue was homogenized in 2 ml of metaphosphoric acid. The resulting homogenate was centrifuged for 10 min at 10,000 g using a refrigerator centrifuge. For total glutathione, 100 μl of the supernatant solution was added to the test tube containing 700 μl of NADPH (0.3 mM), 100 μl of DTNB (6 mM) and 100 μl of distilled water. 10 μl of glutathione reductase was added to the solution after 3-4 min and the absorbance was read at 412 nm. To measure the amount of oxidized glutathione, 100 μl of the centrifuged extract from the previous step was added to a test tube containing 2-vinylpyridine and kept at room temperature for one hr. Then 0.3 mM of NADPH, 6 mM of DTNB and 10 μl of glutathione reductase enzyme were added. The absorption of the samples at 412 nm was read (21).

To prepare the tissue extract for measuring the antioxidant enzymes (catalase, glutathione peroxidase, superoxide dismutase, and glutathione reductase), 0.5 gr of the desired tissue was homogenized in 3 ml of 50 mM phosphate buffer and then the extract was centrifuged in a high-speed refrigerated centrifuge at 10,000 g for 10 min (22).

Catalase (CAT) activity was measured at 240 nm (23). The reaction mixture with a total volume of 3 ml consisted of 50 mM of potassium phosphate buffer with pH = 7, 15 mM of hydrogen peroxide (H ${}_{2}$ O ${}_{2}$ ) and 100 μl of enzyme extract. The amount of H ${}_{2}$ O ${}_{2}$ in the reaction mixture was calculated after one min, which indicated the activity of the enzyme catalase.

Glutathione peroxidase (GPx) activity was measured at 470 nm using guiacol, and the absorption of tetraguiacol which was formed from guiacol as a result of peroxidase activity was measured at 470 nm. The reaction mixture consisted of 2.77 ml of 50 mM phosphate buffer at pH = 7, 100 μl of 1% H ${}_{2}$ O ${}_{2}$ and 100 μl of 4% guiacol (24).

The activity of the superoxide dismutase (SOD) enzyme was measured using the inhibition of NBT optical reduction reaction at 560 nm. The reaction mixture of the sample included 50 mM of phosphate buffer with pH = 7.8, 75 μM of NBT, 0.1 μM of Na-EDTA, 65 μM of riboflavin, 13 mM of methionine and 50 μl of enzyme extract (25).

To measure glutathione reductase (GR) activity, the reaction mixture contained 100 mM of sodium phosphate buffer (pH = 7.8), 0.5 mM of GSSG, 0.1 mM of NADPH and 50 μl of enzyme extract. The reaction was started by adding NADPH. The absorption of the samples was read for three min at a wavelength of 340 nm (26).

To prepare the tissue extract for measuring H ${}_{2}$ O ${}_{2}$ , 0.1 gr of the desired tissue was homogenized in 1 ml of 0.1% trichloroacetic acid and then the extract was centrifuged in a refrigerator centrifuge at a speed of 10,000 g for 10 min. Then 0.5 ml of the centrifuged solution was added to 10 mM of potassium phosphate buffer with pH = 7 and 1 M of potassium iodide, and the absorption was read at 390 nm (27).

### Preparation of testes for histological studies

For histology examination of the testis, the testis was promptly fixed in 10% formaldehyde, buffered by a solution containing 54 mM of NaH ${}_{2}$ PO ${}_{4}$ and 28 mM of Na ${}_{2}$ HPO ${}_{4}$ (pH = 7.4). To complete the tissue fixation, a transverse section was made through the middle of the testis while immersed in the fixator. Thereafter, the tissues were put in paraffin and cut in slices (6-7 micrometer thick) and placed on the glass slides precoated with albumin. The samples were then deparaffinized with xylol, and finally for histological examination by light microscopy (Nikon, Y-THM, Japan) they were stained with hematoxylin and eosin.

Based on the following criteria, a grading score was applied from 1 to 10 for each tubule cross-section. Complete spermatogenesis with perfect tubules = 10, many spermatozoa but disorganized spermatogenesis = 9, a few spermatozoa = 8, absence of spermatozoa but many spermatids = 7, a few spermatids = 6, absence of spermatozoa and spermatids but many spermatocytes = 5, a few spermatocytes = 4, only presence of spermatogonia = 3, absence of germ cells but presence of Sertoli cells = 2, absence of both germ and Sertoli cells = 1.

### Ethical considerations

The study protocol and all animal procedures were approved by the educational assistant of Shahid Bahonar University of Kerman, Kerman, Iran (Code: IR.UK.VETMED.REC.1399.016).

### Statistical analysis 

SPSS (Statistical Package for the Social Sciences) version 20 (from IBM, United States) was used for the statistical analysis. Data are presented as mean ± standard deviation (SD). Statistics were performed by one-way analysis of variance (ANOVA) and Tukey's post hoc test, and significance was considered at p < 0.05.

## 3. Results

### Glucose, testosterone and LH concentrations

**Figure 1:**
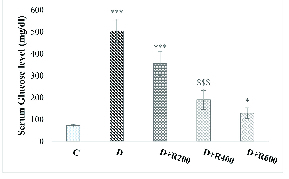
Serum glucose in all groups. *Significant difference (p < 0.05) with D+R400 group. ***Significant difference (p < 0.001) with C, D+R400 and D+R600 groups. ${}^{\text{bad hbox}}$ Significant difference (p < 0.001) with the C group. C: Control, D: Diabetic, D+R: Diabetic + *Rydingia persica*.

The results showed that the glucose concentration was significantly higher in the experimental groups compared to in the C group. The highest concentration was observed in group D and the lowest in group D+R600 (Figure 1).

**Figure 2:**
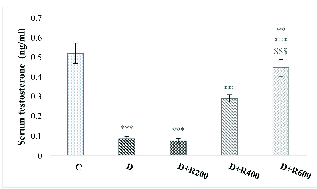
Serum testosterone concentration in all groups. N = 8, Mean ± SD. **Significant difference (p < 0.01) with the C group. ***Significant difference (p < 0.001) with the C group. ${}^{\text{bad hbox}}$ Significant difference (p < 0.001) with the D and D+R200 groups. ${}^{\text{£££}}$ Significant difference (p < 0.001) with the D+R400 group. C: Control, D: Diabetic, D+R: Diabetic + *Rydingia persica*.

**Figure 3:**
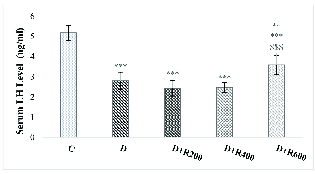
Serum LH concentration in all groups. N = 8, Mean ± SD. **Significant difference (p < 0.01) with the D group. ***Significant difference (p < 0.001) with the C group. ${}^{\text{bad hbox}}$ Significant difference (p < 0.001) with the D+R200 and D+R400 groups. C: Control, D: Diabetic, D+R: Diabetic + *Rydingia persica*, LH: Luteinizing hormone.

Figures 2 and 3 show that serum testosterone and LH concentration were significantly lower in the experimental groups compared to in the C group.

### Effects in the testis tissue

Table I shows that the testis weight in the D and D+R200 groups was significantly lower compared to in the C group. Also, the D+R400 and D+R600 groups showed a significantly higher weight compared to the D and D+R200 groups.

The MDA concentration in the testes in the D, D+R200 and D+R400 groups was also significantly higher compared to in the C group (p < 0.05 and p < 0.01, respectively). However, the D+R600 group showed a significantly lower level compared to the D+R200 group.

The GSH concentration in the testes in the D, D+R200 and D+R400 groups was significantly lower compared to in the C group. Also, the D+R400 and D+R600 groups showed a significantly higher concentration compared to the D and D+R200 groups. Furthermore, the D+R600 group had a significantly higher concentration compared to the D+R400 group.

The GSSG concentration in the testes of the D and D+R200 groups was significantly higher compared to in the C group. Also, the D+R600 group showed a significantly lower concentration compared to the D and D+R200 groups. The D+R400 group had a significantly lower concentration compared to the D+R200 group.

GR activity in the testes of the rats in the D, D+R200, D+R400 and D+R600 groups was significantly lower compared to in the C group. Also, the D+R400 and D+R600 groups showed a significantly higher level compared to the D and D+R200 groups. The D+R600 group showed significantly higher activity compared to the D+R400 group.

GPx activity in the testes of the D, D+R200, D+R400 and D+R600 groups was significantly lower compared to in the C group. Also, the D+R600 rats showed a significantly higher level of activity compared to the D and D+R200 rats.

CAT activity in the testes of the D and D+R200 groups was significantly higher compared to in the C group. Also, the D+R200 group showed a significantly higher level compared to the D+R400 and D+R600 groups.

SOD concentration in the testes of the D, D+R200, D+R400 and D+R600 rats was significantly higher compared to in the C group. The D+R400 and D+R600 groups had a significantly lower concentration compared to the D and D+R200 groups.

H ${}_{2}$ O ${}_{2}$ concentration in the testes in the D, D+R200, D+R400 and D+R600 rats was significantly higher compared to in the C group. Moreover, the D+R600 group had a significantly lower concentration compared to the D, D+R200 and D+R400 groups.

### Effects in the epididymis tissue

Table II shows that the epididymis weight in the D, D+R200 and D+R400 groups was significantly lower compared to in the C group. Also, the D+R600 group showed a significantly higher weight compared to the D and D+R200 groups. The D+R400 group had a significantly higher weight compared to the D and D+R200 groups.

The MDA concentration in the epididymis of the D, D+R200 and D+R400 rats was significantly higher compared to in the C rats, and the D+R600 group's concentration was significantly higher compared to the C group. Also, the D+R600 group had a significantly lower concentration compared to the D, D+R200 and D+R400 groups.

The GSH concentration in the epididymis of the D, D+R200, D+R400 and D+R600 groups was significantly lower compared to in the C group. Also, the D+R600 group showed a significantly higher concentration compared to the D, D+R200 and D+R400 groups.

The GSSG concentration in the epididymis in the D, D+R200, D+R400 and D+R600 groups was a significantly higher level compared to in the C group. Also, the D+R600 and D+R200 groups showed a significantly lower concentration in comparison with the D group.

GR activity in the epididymis of the D, D+R200, D+R400 and D+R600 rats was significantly lower compared to in the C group. Also, the D+R400 and D+R600 rats showed a significantly higher level of activity compared to the D and D+R200 groups, and the D+R600 group demonstrated a significantly higher level compared to the D+R200 group.

GPx concentration in the epididymis of the D, D+R200 and D+R400 groups was significantly lower compared to in the C group. Also, the D+R600 group showed a significantly higher concentration compared to the D, D+R200 and D+R400 groups. The D+R400 group also had a significantly higher concentration compared to the D+R200 group.

CAT activity in the epididymis in the D, D+R200, D+R400 and D+R600 groups was significantly higher compared to in the C group. Also, the D+R400 and D+R600 groups showed significantly higher activity compared to the D+R200 group. The D+R200 and D+R600 groups had significantly lower activity compared to the D group.

SOD activity in the epididymis of the D, D+R200, D+R400 and D+R600 rats was significantly higher compared to in the C group. Also, the D+R600 group showed a significantly lower level compared to the D, D+R200 and D+R400 groups.

H ${}_{2}$ O ${}_{2}$ concentration in the epididymis of the D, D+R200 and D+R400 groups showed a significantly higher level compared to the C group. Also, the D+R600 group had a significantly lower concentration compared to the D, D+R200 and D+R400 groups.

### Histopathology

**Figure 4:**
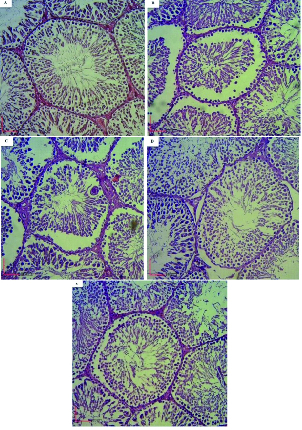
Histopathological examination of the testis. (A) The control group showing the natural structure of the testis. (B) The diabetic group showing structural disorder of the testis. (C) The diabetic group treated with 200 mg/kg *R. persica*, like the diabetic group, showing structural abnormality of the testis. (D & E) Diabetic groups treated with 400 and 600 mg/kg of *R. persica* showing that testicular structure has improved. Magnification: x400.

Figure 4 shows the histopathology of the testis. Examination of the C group did not show spermatogenic arrest, tubular atrophy, edema, or Leydig cell hyperplasia. The estimated Johnson score was about 9/10. The mean of the counted number of germ cells per same sized tubules was 230 per tubule (Figure 4A).

In addition, examination of the D group showed testicular parenchyma with spermatogenic arrest (1+), seminiferous tubular atrophy (3+) and thickening of the basal lamina (2+). It also did not show Leydig cell hyperplasia or edema. The Johnson score was 5/10 basal on the morphological scoring system. The mean of the counted number of germ cells per same sized tubules was 190 per tubule (Figure 4B).

The D+R200 group showed testicular parenchyma with spermatogenic arrest (1+), seminiferous tubular atrophy (2+) and thickening of the basal lamina (2+). It also did not show Leydig cell hyperplasia but moderate interstitial edema was observed (2+). The Johnson score was 5/10 basal on the morphological scoring system. The mean of the counted number of germ cells per same sized tubule was 185.1 per tubule (Figure 4C).

Microscopic examination of the D+R400 group showed testicular parenchyma with spermatogenic arrest (1+), seminiferous tubular atrophy (2+) and thickening of the basal lamina (1+). In this group, Leydig cell hyperplasia was not observed but the examination did show mild interstitial edema (1+). The Johnson score was 6/10 basal on the morphological scoring system. The mean of the counted number of germ cells per same sized tubule was 197.7 per tubule (Figure 4D).

Furthermore, microscopic examination of the D+R600 group did not show spermatogenic arrest or thickening of the basal lamina, but showed atrophy of the seminiferous tubules, Leydig cell hyperplasia, and mild interstitial edema (1+). The Johnson score was 7/10 basal on the morphological scoring system. The mean of the counted number of germ cells per same sized tubule was 212.5 per tubule (Figure 4E).

**Table 1:** Effect of *Rydingia persica *on weight and some of the oxidative and antioxidant factors in the testis tissue of the control and STZ diabetic rats


**Groups**	**C**	**D**	**D+R200**	**D+R400**	**D+R600**
**Testis weight (gr)**	1.23 ± 0.10	0.94 ± 0.06 ${}^{a,\;b,\;c}$	0.97 ± 0.06 ${}^{a,\;b,\;c}$	1.17 ± 0.11	1.17 ± 0.08
**MDA (nm/mg tissue)**	0.230 ± 0.05	0.446 ± 0.10 ${}^{a}$	0.505 ± 0.02 ${}^{a,\;b}$	0.478 ± 0.05 ${}^{a}$	0.295 ± 0.06
**GSH (mg/gr tissue)**	33.07 ± 0.99	17.32 ± 1.27 ${}^{a,\;b,\;c}$	18.78 ± 2.36 ${}^{a,\;b,\;c}$	25.75 ± 1.22 ${}^{a}$	31.13 ± 1.06
**GSSG (mg/gr tissue) **	10.89 ± 0.44	12.95 ± 1.45 ${}^{a,\;b}$	13.63 ± 0.06 ${}^{a,\;b,\;c}$	11.92 ± 0.92	10.78 ± 1.05
**GR (U/mg protein)**	24.22 ± 0.88	14.45 ± 0.96 ${}^{a,\;b,\;c}$	14.53 ± 1.37 ${}^{a,\;b,\;c}$	18.85 ± 1.04 ${}^{a,\;b}$	20.66 ± 1.18 ${}^{a}$
**GPx (U/mg protein)**	0.037 ± 0.008	0.003 ± 0.002 ${}^{a,\;b}$	0.006 ± 0.001 ${}^{a,\;b}$	0.010 ± 0.007 ${}^{a}$	0.020 ± 0.014 ${}^{a}$
**CAT (U/mg protein)**	0.720 ± 0.10	1.170 ± 0.33 ${}^{a,\;b}$	1.500 ± 0.37 ${}^{a,\;b,\;c}$	1.005 ± 0.24	0.084 ± 0.07
**SOD (U/mg protein)**	12.74 ± 0.51	17.31 ± 0.83 ${}^{a,\;b,\;c}$	17.87 ± 0.24 ${}^{a,\;b,\;c}$	14.92 ± 0.92 ${}^{a}$	15.01 ± 0.74 ${}^{a}$
**H_2_O_2_ (nm/m.cm tissue)**	0.51 ± 0.08	3.01 ± 0.14 ${}^{a,\;b}$	2.78 ± 0.23 ${}^{a,\;b}$	2.87 ± 0.17 ${}^{a,\;b}$	2.35 ± 0.48 ${}^{a,\;c}$
Data presented as Mean ± SD. One-way analysis of variance (ANOVA) and Tukey post hoc test for all the variables. Values with different superscripts differ signiﬁcantly: ${}^{a}$ P < 0.05 different from the C group, ${}^{b}$ P < 0.05 different from the D+R600 group, ${}^{c}$ P < 0.05 different from the D+R400 group. C: Control, D: Diabetic, D+R: Diabetic + *Rydingia persica*, MDA: Malondialdehyde, GSH: Glutathione, GSSG: Oxidized glutathione, GR: Glutathione reductase, GPx: Glutathione peroxidase, CAT: Catalase, SOD: Superoxide dismutase, H ${}_{2}$ O ${}_{2}$ : Hydrogen peroxide					

**Table 2:** Effect of *Rydingia persica* on weight and some of the oxidative and antioxidant factors in the epididymis tissue of the control and STZ diabetic rats


**Groups**	**C**	**D**	**D+R200**	**D+R400**	**D+R600**
**Epididymis weight (gr)**	0.507 ± 0.03	0.278 ± 0.02 ${}^{a,\;b}$	0.333 ± 0.02 ${}^{a,\;b}$	0.405 ± 0.06 ${}^{a}$	0.501 ± 0.02
**MDA (nm/mg tissue)**	0.49 ± 0.25	1.98 ± 0.01 ${}^{a,\;b}$	1.76 ± 0.28 ${}^{a,\;b}$	1.76 ± 0.06 ${}^{a,\;b}$	0.79 ± 0.15 ${}^{a}$
**GSH (mg/gr tissue)**	31.07 ± 1.31	15.21 ± 0.88 ${}^{a,\;b}$	14.9 ± 1.07 ${}^{a,\;b}$	18.10 ± 4.80 ${}^{a,\;b}$	23.20 ± 2.72 ${}^{a}$
**GSSG (mg/gr tissue)**	7.81 ± 0.61	11 ± 0.53 ${}^{a,\;b}$	10.02 ± 0.80 ${}^{a,\;d}$	10.35 ± 0.62 ${}^{a}$	9.48 ± 0.56 ${}^{a}$
**GR (U/mg protein)**	22.47 ± 1.31	12.23 ± 0.99 ${}^{a,\;b,\;c}$	12.70 ± 1.00 ${}^{a,\;b,\;c}$	18.10 ± 0.73 ${}^{a,\;b}$	19.87 ± 0.98 ${}^{a}$
**GPx (U/mg protein)**	0.042 ± 0.002	0.013 ± 0.004 ${}^{a,\;b}$	0.010 ± 0.004 ${}^{a,\;b,\;c}$	0.023 ± 0.003 ${}^{a,\;b}$	0.037 ± 0.001
**CAT (U/mg protein)**	0.48 ± 0.02	0.84 ± 0.14 ${}^{a,\;b}$	1.21 ± 0.14 ${}^{a,\;b,\;c}$	0.81 ± 0.08 ${}^{a}$	0.71 ± 0.08 ${}^{a}$
**SOD (U/mg protein)**	9.43 ± 0.53	12.17 ± 0.48 ${}^{a,\;b}$	12.71 ± 0.80 ${}^{a,\;b}$	11.85 ± 0.28 ${}^{a,\;b}$	10.60 ± 0.95 ${}^{a}$
**H_2_O_2_ (nm/m.cm tissue)**	0.64 ± 0.07	0.96 ± 0.14 ${}^{a,\;b}$	1.00 ± 0.17 ${}^{a,\;b}$	0.93 ± 0.03 ${}^{a,\;b}$	0.71 ± 0.08
Data presented as Mean ± SD. One-way analysis of variance (ANOVA) and Tukey post hoc test for all the variables. Values with different superscripts differ signiﬁcantly: ${}^{a}$ P < 0.05 different from the C rats, ${}^{b}$ P < 0.05 different from the D+R600 group, ${}^{c}$ P < 0.05 different from the D+R400 group, ${}^{d}$ P < 0.05 different from the D group. C: Control, D: Diabetic, D+R: Diabetic + *Rydingia persica*, MDA: Malondialdehyde, GSH: Glutathione, GSSG: Oxidized glutathione, GR: Glutathione reductase, GPx: Glutathione peroxidase, CAT: Catalase, SOD: Superoxide dismutase, H ${}_{2}$ O ${}_{2}$ : Hydrogen peroxide					

## 4. Discussion 

In this study, the diabetic group showed increased serum glucose, decreased serum LH and testosterone, decreased testis and epididymis weight, and finally, it showed changes in all variables assayed related to OS, indicating DM-induced OS. Also, diabetic rats treated with R.P with a dose of 600 mg/kg, and in some cases the dose of 400 mg/kg, showed changes in measured variables toward the control group, indicating the protective effect of R.P against OS, testosterone and LH changes.

A previous study emphasized the connection between DM and OS and the resulting complications. DM-induced OS can be the result of increased glucose auto-oxidation from hyperglycemia and protein glycation followed by oxidative degradation of glycated proteins (28). It has been reported that the MDA level in the tissues and blood of diabetic rats is increased due to both lipid peroxidation and reduced antioxidant activity (29, 30). In this study, testis and epididymis MDA levels were significantly higher in the diabetic group. Glutathione plays an important role in cellular defense against ROS. We saw a significant depletion of GSH in the testis and epididymis of diabetic rats, which is a sign of OS (31). In the present study, lower levels of GSH and higher levels of GSSG in the diabetic rats probably were related to the lower activity of GR in both the testis and epididymis of diabetic rats as also described in another study (31). We observed higher activity of SOD and CAT in the diabetic group. As has already been shown, SOD converts superoxide anion to H ${}_{2}$ O ${}_{2}$ which in turn is converted to H ${}_{2}$ O by CAT (32, 33).

Increased activity of CAT in the heart and liver and decreased activity in the kidney of diabetic rats have already been shown (34). In another study, DM led to increased CAT and SOD activity in the liver and serum of diabetic rats (35). Also, it was shown that CAT activity increased in the testis, epididymis and liver of diabetic rats (33, 36). In addition, increased SOD and CAT enzyme expression with hyperglycemia have been demonstrated (37). So, the increased activity of SOD and CAT observed in the present study can be interpreted as a compensatory response against hyperglycemia-induced OS.

We saw decreased serum testosterone in the diabetic group. Leydig cell membrane injury induced by OS can be one explanation for this (15). It can also be the result of steroidogenic defects in the Leydig cells in DM (16). Moreover, the mechanism by which testosterone is reduced in DM may be the direct effect of glucose or its metabolism. It has been reported that insulin stimulates the hypothalamus and hypophysis for secretion of follicle stimulating hormone and LH; therefore, DM leads to a reduction in these two hormones and thereby disturbs spermatogenesis and testosterone production (38). In the present study, diabetic rats treated with R.P with the dose of 400 or 600 mg/kg showed changes in the measured variables toward the control group, indicating the protective effect of R.P against OS. Honey has been shown to reverse the reduced LH and testosterone in diabetic animals toward the control group (39). In our study, the D+R400 group did not display any effects on LH concentration, but showed increased testosterone concentration, while for the D+R600 group, effects both on LH and testosterone were observed. The reason for this could be that at a dose of 600 mg/kg, R.P could have some effect on hypophysis, but at dose of 400 mg/kg, R.P had its effect directly on the testis (40).

The histological examinations of the testis showed that in diabetic rats, spermatogenesis was impaired compared to in control rats. These histological changes included a decreased number of spermatogonia, atrophy of the seminiferous tubules, thickening of the basal lamina, hypoplasia of the Leydig cells, and presence of edema in the diabetic testes. In the present study, the Johnson scores of the diabetic rats were 5, indicating testicular damage. Our results are in agreement with a study that showed DM causes cell death through apoptosis leading to testicular dysfunction, seminiferous tubule atrophy, reduction in tubule diameter, and diminished spermatogenetic cell series (41). Basal lamina thickness has a crucial role in spermatogenesis and its increase results in diminished sperm production and decrease in the size of seminiferous tubules (42). In the histopathological examination of the epididymis, we observed a decrease in sperm density, and atrophy and vacuolation of the epithelium in the diabetic group, which is consistent with another study (43). However, treatment with R.P had a protective effect on the studied tissues, which is consistent with biochemical data.

R.P is used widely by the people of Baluch who live in the southeastern part of Iran. It has some antioxidant, antidiabetic, antimicrobial, anti-inflammatory and antimalarial properties (14). The hypoglycemic effect of R.P may be due to the presence of one or more antidiabetic components which demonstrate synergistic properties (44). Phytochemical screening of the R.P crude extract have revealed the presence of flavonoids, phenolic acids, and steroids/ triterpenoids (44, 45).

Flavonoids have been shown to work as antidiabetic compounds, because they possess multiple characteristics that promote both glucose-lowering actions (insulin-mimetic) and insulin secretion (insulin secretagogue) (46). Two flavonoids, morin and quercetin, both present in R.P, have been shown to have strong antioxidant activity, which is equal to BHA and stronger than alpha tocopherol (47). Quercetin strongly inhibits the transport of glucose and fructose through intestinal cell GLUT2 (48). Another study showed it potentiates insulin secretion induced by glucose and has a protective effect against beta-cell oxidative injury induced by H ${}_{2}$ O ${}_{2}$ (49). The effects of quercetin administrated intraperitoneally was analyzed (10 or 15 mg/kg body weight) for 10 days in control and diabetic rats which significantly decreased glucose levels in the diabetic rats. Quercetin increases the glucokinase activity, leading to this effect (50). Also it has been shown that quercetin has a similar effect as metformin (51).

The antioxidant activity of morin has been reported to be effective against OS in several disease profiles (52-54). Administration of morin has been reported to improve the pathological condition of hyperglycemia, glucose intolerance, lipid peroxidation, and insulin resistance (52, 55). Improvement in insulin receptor signaling and reduction of hyperglycemia and lipid deposits in the liver of diabetic rats has been reported, as well as antidiabetic and antioxidant effects, which directly supports the notion of the antidiabetic activity of morin (56, 57). In another study, morin administration was shown to increase insulin receptor activation and decrease gluconeogenesis potency (58). The effect of morin has been found to be similar to the conventionally used antidiabetic drug Glibenclamide. Moreover, monoterpene derivatives in the R.P flower are other compounds with antioxidant activity (59).

Therefore, it can be suggested that the reason for the improvement in the hyperglycemic, oxidative, and antioxidant status observed in the rats treated with R.P powder was due to the insulin-like activity and antidiabetic properties of R.P.

## 5. Conclusion

Our findings showed that R.P powder in doses of 600 or 400 mg/kg body weight had protective effects against DM by reducing hyperglycemia, defending against OS, and restoring hormonal and testicular histological changes.

##  Conflict of Interest

The authors declare that there is no conflict of interest.
